# The importance of minimizing carbon footprint in Ophthalmology to ensure environmental sustainability

**DOI:** 10.22336/rjo.2024.17

**Published:** 2024

**Authors:** Consuela-Mădălina Gheorghe

**Affiliations:** * Philologist, Certified translator

Being one of the most important problems worldwide, reducing the carbon footprint in ophthalmology should be addressed properly by each nation. According to the website https://www.nature.org/en-us/get-involved/how-to-help/carbon-footprint-calculator/, carbon footprint represents the total amount of greenhouse gases generated by actions of human beings. The average carbon footprint is about 4 tons globally, and it should drop to under 2 tons to be in the proper parameters. Thus, to diminish the carbon footprint, the community should be educated regarding more sustainable practices, how to reduce the carbon footprint, and surgical waste, and support research and innovation in ophthalmological science, and its environmental impact. 

The health care industry’s greenhouse gas emissions are shocking. 4.4% of greenhouse gas emissions worldwide are attributable to the health care sector, with 71% coming from the medical supplies’ production, use, and disposal. According to Sarishka Desai, BA, from the University of Connecticut School of Medicine in Farmington, and colleagues, ophthalmology performs more procedures than any other medical profession, with 50 million cataract surgeries estimated to be carried out worldwide by 2050 (https://www.ophthalmologytimes.com/view/minimizing-the-carbon-footprint-of-ophthalmic-surgeries).

In this vein, it is believed that there is an enormous amount of waste generated as a result of the procedures in the operating rooms, hence it is important to find and use waste reduction strategies. Recycling and proper waste sorting have a positive and financial impact. In addition, a recycling improvement program consisting of training sessions for the staff and educational materials is of paramount importance for environmental sustainability (Sherry B, Lee S, De Los Angeles Ramos Cadena M, Laynor G, Patel SR, della Badia Simon M, Romanowski EG, Hochman SE, Schuman JS, Prescott C, Thiel CL. How Ophthalmologists Can Decarbonize Eye Care: A Review of Existing Sustainability Strategies and Steps Ophthalmologists Can Take. Ophthalmology. 2023; 130(7):702-714. https://doi.org/10.1016/j.ophtha.2023.02.028). At the same time, measures should be taken to reduce the single-use instruments by applying more immediate use steam sterilization, alcohol-based scrub instead of water-based scrub, etc., and disposable gowns and drapes for eye surgeries should be used only when needed.

Intravitreal injections have become one of the most common procedures in ophthalmology over the years (Cameron TW 3rd, Vo LV, Emerson LK, Emerson MV, Emerson GG. Medical Waste Due to Intravitreal Injection Procedures in a Retina Clinic. J Vitreoretin Dis. 2021 Feb 10;5(3):193-198. doi: 10.1177/2474126420984657. PMID: 37006514; PMCID: PMC9979047, cited by March de Ribot A and March de Ribot F). Regarding potential solutions and future directions to reduce the carbon footprint, it is possible to optimize the intravitreal injections, so that patients can safely have the injections and consultations on the same day, thus being the most practical method for them and the ophthalmologist. At the same time, travel costs and travel-associated emissions could be decreased by scheduling fewer follow-up visits for cases that are not complicated. Other ways to reduce carbon emissions imply not opening so many items from packs and using them rationally, meaning without throwing away items that were opened and not used.

Moreover, according to the same study by Cameron TW et al. cited by March de Ribot A and March de Ribot F, cold packs, cardboard boxes, foam coolers, and nitrile gloves seem to have contributed the most to the overall waste, among which the non-biodegradable foam coolers being the ones that had the greatest environmental impact, implying high CO2 emissions. 

Regarding cataract surgeries, the results of a study published by Morris DS et al. (Morris DS, Wright T, Somner JE, Connor A. The carbon footprint of cataract surgery. Eye (Lond). 2013 Apr;27(4):495-501. doi: 10.1038/eye.2013.9. Epub 2013 Feb 22. PMID: 23429413; PMCID: PMC3626018) underlined that the carbon footprint for only one such operation was 181.8 kg CO2eq. Taking this into consideration, it is apparent that hundreds if not thousands of carbon footprint tones CO2eq are produced by each ophthalmological clinic that provides cataract surgeries, without calculating the emissions attributable to the building energy use, the travel of the patient and staff and the procurement of pharmaceuticals, medical equipment, etc.

According to Mukamal R, who underwent a study titled “Sustainability in Ophthalmology. Going green for the future of eye care”, waste also seems to be happening at the medication use level. A fresh bottle of eye drops is discarded after only one use in many ophthalmic operations, even in cases where the patient needs a prescription for the same medication after being released from the hospital. Thus, this kind of waste seems to generate emissions that are equivalent to approximately 22,624 gasoline-powered cars or the electricity use of 20,430 homes for one year (Taboun O et al. Cataract Refract Surg. 2023;49(7):759-763 cited by Mukamal R. Sustainability in Ophthalmology. Going green for the future of eye care. Nov 01, 2023. Eyenet Magazine. 45-51).

In conclusion, ophthalmologists should work to mitigate and adapt to the health threats posed by the carbon footprint. Several studies have also been undertaken to underline the importance of reducing the carbon footprint, but more are needed to shift policies. Furthermore, measures and instruments must be created to calculate and track the carbon emissions from eye care services.


**Assist. Prof. Consuela-Mădălina Gheorghe, PhD, Philologist, Certified translator**


